# Mathematical analysis of a nutrient–plankton system with delay

**DOI:** 10.1186/s40064-016-2435-7

**Published:** 2016-07-11

**Authors:** Mehbuba Rehim, Zhenzhen Zhang, Ahmadjan Muhammadhaji

**Affiliations:** College of Mathematics and System Sciences, Xinjiang University, Urumqi, 830046 Xinjiang China

**Keywords:** Toxic phytoplankton, Time delay, Stability, Hopf-bifurcation, Nutrient recycling

## Abstract

A mathematical model describing the interaction of nutrient–plankton is investigated in this paper. In order to account for the time needed by the phytoplankton to mature after which they can release toxins, a discrete time delay is incorporated into the system. Moreover, it is also taken into account discrete time delays which indicates the partially recycled nutrient decomposed by bacteria after the death of biomass. In the first part of our analysis the sufficient conditions ensuring local and global asymptotic stability of the model are obtained. Next, the existence of the Hopf bifurcation as time delay crosses a threshold value is established and, meanwhile, the phenomenon of stability switches is found under certain conditions. Numerical simulations are presented to illustrate the analytical results.

## Background

Plankton refers to organisms that are living in water bodies (such as oceans, lakes, rivers and ponds) freely drifting and weakly mobile (Abdllaoui et al. 2002; Odum 1971). Plant forms of plankton community are known as phytoplankton, they serve as the basic food source and occupy the first trophic level of all aquatic food chains. Animals in the plankton community are known as zooplankton. They consume phytoplankton which are their most favourable food source. Phytoplankton are not only the basis for all aquatic food chains, but also they do huge services for our earth by supplying the essential oxygen and absorbing the harmful carbon dioxide which contributes to global warming (Odum 1971). In addition to these benefits phytoplankton act as the biological indicators of water quality. Excess blooming of the phytoplankton will deteriorate the water quality. For example, increase of phytoplankton population in lakes (reservoirs), especially the extension of the growing season and over growing of the cyanobacteria are important causes for eutrophication in lakes. Eutrophication refers to the enrichment of an ecosystem with chemical nutrients such as nitrogen, phosphate and so on, leading to the over growth of biomass and their rapid reproduction in water bodies. This leads to the decrease of dissolved oxygen in water, which in turn results in the death of aquatic organisms. These dead aquatic organisms get settled at the bottom of the lake and then decomposed by microorganisms which once again consume a large amount of dissolved oxygen. Consequently, the dissolved oxygen content of the water body is further reduced and the water quality deteriorates further. This process affects the survival of aquatic organism and greatly accelerates the process of eutrophication in water bodies. The occurrence of the eutrophication, because of a large amount of reproduction of plankton, often makes the water bodies appear in different colors such as blue, red, brown, white, and so on. This phenomenon occurring in water bodies is called “algae bloom” and “red tide” in sea. These algae are foul smelling, poisonous and can’t be eaten by fish. And also they prevent sunlight from reaching the submerged plants and leading to their death by hindering their photosynthesis. These dead submerged plants releasing nitrogen, phosphorus and other nutrients after decaying and then the algae use these nutrients. Because of the high biomass accumulation or the presence of toxicity, some of these blooms, more adequately called “harmful algal blooms” (Smayda 1997), are noxious to marine ecosystems or to human health and can produce great socioeconomic damage. Therefore, the study of marine plankton ecology is an important consideration for the survival of our earth.

Due to the difficulty of measuring plankton biomass, mathematical modeling of plankton population is an important alternative method of improving our knowledge of the physical and biological processes relating to plankton ecology (Edwards and Brindley 1999). The problems of zooplankton–phytoplankton systems have been discussed by many authors (Rose 2012; Saha and Bandyopadhyay 2009; Chakraborty and Dasb 2015; Yunfei et al. 2014; Rehim and Imran 2012; Ruan 1995) in resent years. These systems can exhibit rich dynamic behavior, such as stability of equilibria, Hopf bifurcation, global stability, global Hopf bifurcation and so on. However, the importance of nutrients to the growth of plankton leads to explicit incorporation of nutrients concentrations in the phytoplankton–zooplankton models. Therefore, a better understanding of mechanisms that determine the plankton is to consider plankton–nutrient interaction models. Recently, a nutrient–plankton model system for a water ecosystem is proposed by Fan et al. (2013) and its global dynamics behavior under different levels of nutrient has been studied. He and Ruan (1998), Zhang and Wang (2012), Pardo (2000) studied nutrient–phytoplankton interaction model and observed the global behavior of the system. Huppert et al. (2004) studied a simple nutrient–phytoplankton model to explore the dynamics of phytoplankton bloom. Huppert et al. (2004) provided a full mathematical investigation of the effects of three different features in an excitable system framework.

The understanding of the dynamic of plankton–nutrient system becomes complex when additional effects of toxicity (caused due to the release of toxin substances by some phytoplankton species known as harmful phytoplankton) are considered. The role of toxin and nutrient on the plankton system have been discussed by many researchers (Chakarborty et al. 2008; Pal et al. 2007; Khare et al. 2010; Jang et al. 2006; Chowdhury et al. 2008; Upadhyay and Chattopadhyay 2005; Chatterjee et al. 2011).

Time delays of one type or another have been incorporated into biological models by many researchers (Aiello and Freedman 1990; Chen et al. 2007; Cooke and Grossman 1982; Hassard et al. 1981; Song et al. 2004). In general, delay differential equations exhibit much more complicated dynamics than ordinary differential equations since a time delay could cause a stable equilibrium to become unstable and induce oscillations and periodic solutions. Therefore, more realistic models of population interactions should take into account the effect of time delays. The interaction of plankton–nutrient model with delay due to gestation and nutrient recycling has been studied by Ruan (1995) and Das and Ray (2008). Chattopadhyay et al. (2002) proposed and analysed a mathematical model of toxic phytoplankton–zooplankton interaction and assumed that the liberation of toxic substances by the phytoplankton species is not an instantaneous process but is mediated by some time lag required for maturity of species. Extending the work of Chattopadhyay et al. (2002), Saha and Bandyopadhyay (2009) and Rehim and Imran (2012) have studied the global stability of the toxin producing phytoplankton–zooplankton system.

The effect of nutrient recycling on food chain dynamics has been extensively studied. Nisbet et al. (1983), Ruan (1993), Angelis (1992) and Ghosh and Sarkar (1998) studied the effect of nutrient recycling for ecosystem. In their model the nutrient recycling is considered as an instantaneous process and the time required to regenerate nutrient from dead organic is neglected. Beretta et al. (1990), Bischi (1992) and Ruan (2001) studied nutrient recycling model with time delay. They have performed a stability and bifurcation analysis of the system and estimated an interval of recycling delay that preserves the stability switch for the model.

In the present paper, motivated by the above work, a model for the nutrient–plankton consists of dissolved nutrient (N), phytoplankton (p) and herbivorous zooplankton (z) is considered. We assume that the functional form of biomass conversion by the zooplankton is Holling type-II and the predator is obligated that is they dose not take nutrient directly. The toxic substance term which induces extra mortality in zooplankton is also expressed by Holling type II functional form.

In order to account for the time needed by the phytoplankton to mature after which they can release toxins, a discrete time delay is incorporated into the system. Moreover, the discrete delays also indicate the partially recycled nutrient decomposed by bacteria after the death of biomass. The models in Fan et al. (2013)and Das and Ray (2008), time required to regenerate nutrient from dead organisms is neglected. Also the term of toxin liberation has not take into their model. But these are one of the most important features in the real ecosystem (Sarkara et al. 2005; Chattopadhayay et al. 2002; Mukhopadhyay and Bhattacharyya 2010). In comparison with literature (Fan et al. 2013; Das and Ray 2008), the model proposed in this paper is more general and realistic.

The organization of the paper is as follows. In next section, a nutrient–plankton delay differential equations with delay will be proposed and its boundedness criteria will be established. In “Equilibria and its stability” section, we analyze the dynamical properties such as existence of the equilibria and its stability, possible bifurcations with variation of the parameters. In “Numerical simulation” section, numerical studies of the models are performed to support our analytical results. Discussion are drawn in the final section.

## The model

Let *N*(*t*), *p*(*t*) and *z*(*t*) are the concentration of nutrient, phytoplankton and zooplankton population at time *t*, respectively. Let $N_0$ be the constant input of nutrient concentration and *D* be the washout rates for nutrient, phytoplankton and zooplankton, respectively. The constant delay parameter $\tau _1$, $\tau _2$ and $\tau _3$ are considered in the decomposition of phytoplankton population, zooplankton population and the discrete time period required for the maturity of toxic-phytoplankton, respectively. With these assumptions, we write the following system of delay differential equations describing nutrient–plankton interaction

1 $$\left\{ \begin{array}{ll} \displaystyle \frac{dN}{dt}=D(N_0-N(t))-\alpha N(t)p(t)+m_1d_1p(t-\tau _1)+m_2d_2z(t-\tau _2),\\ \displaystyle \frac{dp}{dt}=k_1\alpha N(t)p(t)-(D+d_1)p(t)-\frac{\beta p(t)z(t)}{a+p(t)}-\upsilon p^2(t),\\ \displaystyle \frac{dz}{dt}=\frac{k_2\beta p(t)z(t)}{a+p(t)}-(D+d_2)z(t)-\frac{k_3\beta p(t-\tau _3)z(t)}{a+p(t-\tau _3)}.\\ \end{array} \right.$$

We assume that all parameters are non-negative and are interpreted as follows:

- $\alpha$—nutrient uptake rate for the phytoplankton
- $\beta$—the maximum zooplankton ingestion rate
- $d_1$—the natural death rate of phytoplankton
- $d_2$—the natural death rate of zooplankton
- $m_1$—the nutrient recycle rate after the death of phytoplankton population $0<m_1<1$
- $m_2$—the nutrient recycle rate after the death of zooplankton population $0<m_2<1$
- $k_1$—the conversion factor from nutrient to phytoplankton $0<k_1<1$
- $k_2$—the conversion factor from phytoplankton to zooplankton $0<k_2<1$
- $k_3$—the rate of toxic substances produced by per unit biomass of phytoplankton
- *a*—the half saturation constant
- $\upsilon$—the intra-specific competition coefficient or the density dependent mortality rate of phytoplankton population.

- As pointed out by Holling (1965), Ma (1996) and Das and Ray (2008), the functional response of Holling type I is applied to lower organisms, for example, alga and unicellular organism. Therefore, in this paper we let the functional response of phytoplankton to nutrient be Holling type I.
- As phytoplankton is the most favorable food source for zooplankton within aquatic environments and the Holling type-II functional form is a reasonable assumption to describe the law of predation (Chattopadhyay et al. 2002; Ludwig et al. 1978; Das and Ray 2008). It is quite reasonable to assume that the law of grazing must be same whether it contributes toward the growth of zooplankton species or it suppresses the rate of grazing due to presence of toxic substances.
- In fact, the liberation of toxic substance by phytoplankton is not an instantaneous phenomenon, since it must be mediated by some time lag which is required for the maturity of toxic-phytoplankton. However, the liberation of toxic substance at the time t depends on the population size of toxic phytoplankton species at time $t-\tau _3$. So, the zooplankton mortality due to the toxic phytoplankton is described by the term $p(t-\tau _3)z(t)$. In model (1), the term $\frac{\rho p(t-\tau _3)z(t)}{a+p(t-\tau _3)}$ describe the distribution of toxic substance which ultimately contributes to the death of zooplankton populations.
- Our model consider that the phytoplankton have competition among themselves for their survival (Barton and Dutkiewicz 2010; Jana et al. 2012; Ruan et al. 2007; Wang et al. 2014). $\upsilon p^2$ is the reduction term for the phytoplankton population.

Here we observe that, if there is no delay (i.e., $\tau _i=0$ ) and $k_2<k_3$, then $\dot{z}<0$. If $k_2>k_3$ and $\beta (k_2-k_3)-(D+d_2)<0$, then we also get $\dot{z}<0$. Hence, throughout our analysis, we assume that

$$\begin{aligned} \beta (k_2-k_3)-(D+d_2)>0. \end{aligned}$$

From the standpoint of biology, we are only interested in the dynamics of model (1) in the closed first octant $R^3_+$.

In accordance with the biological meaning, the initial conditions of the system (1) are taken as follows

2 $$\begin{aligned} N(0)\ge 0, \quad p(\theta )\ge 0, \quad z(\theta )\ge 0 \quad \text{ for}\; \theta \in [-\tau , 0]. \end{aligned}$$

where $$\tau =\max \{\tau _1, \tau _2, \tau _3\}$$.

Regarding the positivity and boundedness of the solution for the system (1) we state the following theorem.

### **Theorem 1**

*All solutions of system* (1) *with initial conditions* (2) *are positive and bounded.*

### *Proof*

The proof of positivity of the solutions of system (1) is easy, so we omit it here. As for boundedness of the solutions of (1), we define function

$$X(t)=N(t)+\frac{p(t)}{k_1}+\frac{z(t)}{k_1(k_2-k_3)}.$$

Derivative of X(t) with respect to system (1), we obtain

$$\begin{aligned} X^{'}(t)&= D\left( N_0-N-\frac{p(t)}{k_1}-\frac{z(t)}{k_1(k_2-k_3)}\right) -\frac{\upsilon }{k_1}p^2-\frac{d_1(1-m_1k_1)}{k_1}p-\frac{d_2(1-m_2k_1(k_2-k_3))}{k_1(k_2-k_3)}z\\&< D(N_0-X). \end{aligned}$$

Therefore, $X<N_0+\epsilon$ for all large t, where $\epsilon$ is an arbitrarily small positive constant. Thus, N(t), p(t) and z(t) are ultimately bounded by some positive constant. $\square$

## Equilibria and its stability

### Equilibria

System (1) possesses three possible nonnegative equilibria, namely the extinction equilibrium $E_0(N_0,0,0)$, the zooplankton-eradication equilibrium $E_1(N_1^{*},p_1^{*},0)$ and the coexistence equilibrium $E^{*}(N^{*},p^{*},z^{*})$. For the zooplankton-eradication equilibrium $E_1(N_1^{*},p_1^{*},0)$, the $N_1^{*}$ and $p_1^{*}$ satisfy the following equation:

3 $$\left\{ \begin{array}{ll} \displaystyle DN_0-DN-\alpha Np+m_1d_1p=0,\\ \displaystyle k_1\alpha N-(D+d_1)-\upsilon p=0.\\ \end{array} \right.$$

From first equation of system (3) we have

4 $$N=\frac{DN_0+m_1d_1p}{D+\alpha p}.$$

On substituting (4) into second equation of (3) we derive that

5 $$\begin{aligned} \frac{1}{D+\alpha p}\left[ \alpha \upsilon p^{2}+(D\upsilon +\alpha (D+d_1)-k_1\alpha m_1d_1)p+D(D+d_1-k_1\alpha N_0)\right] =0. \end{aligned}$$

If $N_0>\frac{D+d_1}{k_1\alpha }$, then Eq. (5) has exactly one positive real root

$$\begin{aligned} p_1^{*}=\frac{-[D\upsilon +\alpha (D+d_1)-k_1\alpha m_1d_1]+\sqrt{\Delta }}{2\alpha \upsilon }>0, \end{aligned}$$

where $$\Delta =[D\upsilon +\alpha (D+d_1)-k_1\alpha m_1d_1]^{2}-4\alpha \upsilon D(D+d_1-k_1\alpha N_0)$$.

For the coexistence equilibrium $E^{*}(N^{*},p^{*},z^{*})$, the $N^{*}$, $p^{*}$ and $z^{*}$ satisfy the following equation:

6 $$\left\{ \begin{array}{ll} \displaystyle DN_0-DN-\alpha Np+m_1d_1p+m_2d_2z=0,\\ \displaystyle k_1\alpha N-(D+d_1)-\frac{\beta z}{a+p}-\upsilon p=0,\\ \displaystyle \frac{(k_2-k_3)\beta p}{a+p}-(D+d_2)=0.\\ \end{array} \right.$$

From third equation of system (6) we have

$$p^{*}=\frac{a(D+d_2)}{\beta (k_2-k_3)-(D+d_2)}>0.$$

Again from first and second equations we have

$$\begin{aligned} \left(k_1\alpha m_2d_2-\frac{\beta (D+\alpha p)}{a+p}\right)z= \alpha \upsilon p^{2}+[D\upsilon +\alpha (D+d_1) -k_1\alpha m_1d_1]p+D(D+d_1-k_1\alpha N_0). \end{aligned}$$

Let $f(p)=\alpha \upsilon p^{2}+[D\upsilon + \alpha (D+d_1)-k_1\alpha m_1d_1]p+D(D+d_1-k_1\alpha N_0)$. If $N_0>\frac{D+d_1}{k_1\alpha }$, $\frac{ak_1\alpha m_2d_2}{\beta }<D <a\alpha$ and $0<p^*<p_1^{*}$, then $k_1\alpha m_2d_2-\frac{\beta (D+\alpha p^*)}{a+p^*}<0$ and $f(p^*)<0$. Thus

$$\begin{aligned} z^*&= \frac{a(k_2-k_3)}{(k_2-k_3)(ak_1\alpha m_2d_2-D\beta ) +(D+d_2)(D-a\alpha )}f(p^*)>0,\\ N^*&= \frac{DN_0+m_1d_1p^*+m_2d_2z^*}{D+\alpha p^*}>0. \end{aligned}$$

From above analysis we obtain the following theorem.

#### **Theorem 2**

*The extinction equilibrium*$E_0(N_0,0,0)$*always exists. Furthermore, suppose that*$N_0>\frac{D+d_1}{k_1\alpha }$. *Then the zooplankton-eradication equilibrium*$E_1(N_1^{*},p_1^{*},0)$*exists, and the unique coexistence equilibrium*$E^{*}(N^{*},p^{*},z^{*})$*exists only if*$\frac{ak_1\alpha m_2d_2}{\beta }<D<a\alpha$*and*$0<p^*<p_1^{*}$.

In what follows, we will analysis the stability of the system (1) around different equilibria.

### Model (1) without delay

In this subsection, we give the basic dynamical behavior of system (1) without delay.

#### **Theorem 3**

*(i)*: *If*$N_0<\frac{D+d_1}{k_1\alpha }$, *then the extinction equilibrium*$E_0(N_0,0,0)$*is locally asymptotically stable and*$E_0$*unstable if*$N_0>\frac{D+d_1}{k_1\alpha }$.
*(ii)*: *Suppose that*$N_0>\frac{D+d_1}{k_1\alpha }$. *If*$p_1^*<\frac{a(D+d_2)}{(k_2-k_3)\beta -(D+d_2)}$, *then thezooplankton-eradication equilibrium*$E_1(N_1^{*},p_1^{*},0)$*is locally asymptotically stable and*$E_1$*unstable if*$p_1^*>\frac{a(D+d_2)}{(k_2-k_3)\beta -(D+d_2)}$.
*(iii)*: *Suppose that the coexistence equilibrium*$E^{*}(N^{*},p^{*},z^{*})$*exists. Then it is locally asymptotically stable if the following inequality hold*
  7 $$\begin{aligned} \upsilon>\frac{\beta z^*}{(a+p^*)^2},\quad \alpha N^*-m_1d_1>0\quad and \quad \frac{\beta (D+\alpha p^*)}{a+p^*}>k_1\alpha m_2d_2, \end{aligned}$$

#### *Proof*

The characteristic equation about $E_0(N_0,0,0)$ is given by

8 $$(\lambda +D)(\lambda -k_1\alpha N_0+(D+d_1))(\lambda +D+d_2)=0.$$

It is clear that Eq. (8) has negative root $\lambda _1=-D<0$ and $\lambda _2=-(D+d_2)<0$. So, if $N_0<\frac{D+d_1}{k_1\alpha }$, then

$$\lambda _3=k_1\alpha N_0-(D+d_1)<0.$$

From this we have that the extinction equilibrium $E_0(N_0,0,0)$ is locally asymptotically stable. If $N_0>\frac{D+d_1}{k_1\alpha }$ , then $E_0$ is unstable.

The characteristic equation about $E_1(N_1^{*},p_1^{*},0)$ is $[\lambda -(\frac{(k_2-k_3)\beta p_1^*}{a+p_1^*}-(D+d_2))][\lambda ^2-(k_1\alpha N_1^*-(D+d_1)-2\upsilon p_1^*-D-\alpha p_1^*)\lambda -(D+\alpha p_1^*)(k_1\alpha N_1^*-(D+d_1)-2\upsilon p_1^*)+k_1\alpha p_1^*(\alpha N_1^*-m_1d_1)]=0$. If $p_1^*<\frac{a(D+d_2)}{(k_2-k_3)\beta -(D+d_2)}$, then $\lambda _1=\frac{(k_2-k_3)\beta p_1^*}{a+p_1^*}-(D+d_2)<0.$ Further, $\lambda _2\lambda _3=2\alpha \upsilon p_1^{*^2}+D(\alpha +\upsilon )p_1^*+d_1\alpha p_1^*(1-k_1m_1)>0$ and $\lambda _2+\lambda _3=k_1\alpha N_1^*-(D+d_1)-2\upsilon p_1^*-D-\alpha p_1^*=-\upsilon p_1^*-D-\alpha p_1^*<0$. Which implies that $\lambda _2<0$ and $\lambda _3<0$. Therefore, if $p_1^*<\frac{a(D+d_2)}{(k_2-k_3)\beta -(D+d_2)}$, then the zooplankton-eradication equilibrium $E_1(N_1^{*},p_1^{*},0)$ is locally asymptotically stable and $E_1$ unstable if $p_1^*>\frac{a(D+d_2)}{(k_2-k_3)\beta -(D+d_2)}$.

The characteristic equation about $E^{*}(N^{*},p^{*},z^{*})$ is given by

9 $$\lambda ^{3}+A\lambda ^{2}+B\lambda +C=0,$$

where

$$\begin{array}{ll} A=(\upsilon +\alpha )p^*+D-\frac{\beta p^*z^*}{(a+p^*)^2},\\ B=k_1\alpha p^*(\alpha N^*-m_1d_1)-(D+\alpha p^*)\left( \frac{\beta p^*z^*}{(a+p^*)^2}-\upsilon p^*\right) +\frac{a(k_2-k_3)\beta ^2 p^*z^*}{(a+p^*)^3}, \\ C=\frac{a(k_2-k_3)\beta z^{*}}{(a+p^{*})^2}\left[ \frac{\beta p^*}{a+p^*}(D+\alpha p^*)-k_1\alpha m_2d_2p^*\right] . \end{array}$$

If $\upsilon >\frac{\beta z^*}{(a+p^*)^2}$, $\alpha N^*-m_1d_1>0$ and $\frac{\beta (D+\alpha p^*)}{a+p^*}>k_1\alpha m_2d_2$, then

$$\begin{aligned} A&= (\upsilon +\alpha )p^*+D-\frac{\beta p^*z^*}{(a+p^*)^2}>0;\\ B&= k_1\alpha p^*(\alpha N^*-m_1d_1)-(D+\alpha p^*)\left( \frac{\beta p^*z^*}{(a+p^*)^2}-\upsilon p^*\right) +\frac{a(k_2-k_3)\beta ^2 p^*z^*}{(a+p^*)^3}>0;\\ C& = \frac{a(k_2-k_3)\beta z^{*}}{(a+p^{*})^2}\left[ \frac{\beta p^*}{a+p^*}(D+\alpha p^*)-k_1\alpha m_2d_2p^*\right]>0.\\ AB-C&= \left[ (\upsilon +\alpha )p^*+D-\frac{\beta p^*z^*}{(a+p^*)^2}\right] \left[ k_1\alpha p^*(\alpha N^*-m_1d_1)-(D+\alpha p^*)\left( \frac{\beta p^*z^*}{(a+p^*)^2}-\upsilon p^*\right) \right. \\& \quad+\left.\frac{a(k_2-k_3)\beta ^2 p^*z^*}{(a+p^*)^3}\right] -\frac{a(k_2-k_3)\beta z^{*}}{(a+p^{*})^2}\left[ \frac{\beta p^*}{a+p^*}(D+\alpha p^*)-k_1\alpha m_2d_2p^*\right] \\&= k_1\alpha p^*(\alpha N^*-m_1d_1)(D+\alpha p^*)+(D+\alpha p^*)^2 \left(\upsilon p^*-\frac{\beta p^*z^*}{(a+p^*)^2}\right) \\&\quad+\left( \upsilon p^*- \frac{\beta p^*z^*}{(a+p^*)^2}\right) k_1\alpha p^*(\alpha N^*-m_1d_1)+(D+\alpha p^*)\left( \frac{\beta p^*z^*}{(a+p^*)^2}-\upsilon p^*\right) ^2 \\&\quad+\left( \upsilon p^*- \frac{\beta p^*z^*}{(a+p^*)^2}\right) \frac{a(k_2-k_3)\beta ^2 p^*z^*}{(a+p^*)^3}+\frac{a(k_2-k_3)k_1\alpha \beta m_2d_2p^*z^*}{(a+p^*)^2}\\&>0. \end{aligned}$$

Therefore, all roots of (9) have negative real parts. By the Routh–Hurwitz criterion we obtain that the coexistence equilibrium $E^{*}( N^{*}, p^{*}, z^{*})$ is locally asymptotically stable. $\square$

#### Remark 1

From above analysis we see that the input concentration of the nutrient, density dependent mortality rate of phytoplankton population and the death rate of the plankton play an important role in controlling the dynamics of the system.

After studying the local stability behavior we perform a global analysis around the equilibrium point.

#### **Theorem 4**

*If*$k_1\le \min \{\frac{D+d_1}{m_1d_1},\frac{D+d_2}{(k_2-k_3)m_2d_2}\}$, *then the extinction equilibrium*$E_0(N_0,0,0)$*is globally asymptotically stable.*

#### *Proof*

Define a positive definite function

$$V_0=N-N_0-N_0\ln\frac{N}{N_0}+\frac{1}{k_1}p+ \frac{1}{k_1(k_2-k_3)}z.$$

Calculating the derivative of $V_0$ along the positive solution of system (1) we have

$$\begin{aligned} \frac{dV_0}{dt}\Big |_{(1)} &=\frac{N-N_0}{N}\dot{N}+\frac{1}{k_1}\dot{p}+\frac{1}{k_1(k_2-k_3)} \dot{z}\\ &=-\frac{D(N-N_0)^2}{N}-\frac{\upsilon }{k_1}p^2+\left( m_1d_1-\frac{D+d_1)}{k_1}\right) p-\frac{N_0m_1d_1}{N}p+\left( m_2d_2-\frac{D+d_2}{k_1(k_2-k_3)}\right) z\\&-\frac{N_0m_2d_2}{N}z. \end{aligned}$$

Since N(t), p(t) and z(t) are positive, if $k_1\le \min \{\frac{D+d_1}{m_1d_1},\frac{D+d_2}{(k_2-k_3)m_2d_2}\}$, then $\frac{dV_0}{dt}\Big |_{(1)}\le 0$. $\frac{dV_0}{dt}\Big |_{(1)}=0$ if and only if $(N,p,z)=(N_0,0,0)$. Thus $E_0$ is globally asymptotically stable by Lyapunov–LaSalle invariance principle. $\square$

#### Remark 2

Theorem 4 shows that too low of the conversion rate of the plankton will cause species extinction. This is consistent with the real ecosystem.

For the globally asymptotically stability of the equilibrium $E_1(N_1^{*},p_1^{*},0)$, we first consider the transformations $N=N_1^*+N_1$, $p=p_1^*+p_1$, $z=z_1$. With these transformations, the model (1) reduces to

10 $$\left\{ \begin{array}{ll} \displaystyle \frac{dN_1}{dt}=-DN_1-\alpha (N_1p_1+N_1^*p_1+N_1p_1^*) +m_1d_1p_1+m_2d_2z_1,\\ \displaystyle \frac{dp_1}{dt}=k_1\alpha (N_1p_1+N_1^*p_1+N_1p_1^*) -(D+d_1)p_1-\frac{a\beta p_1z_1}{(a+p_1^*)^2}-\frac{\beta p_1^*z_1}{a+p_1^*}-2\upsilon p_1^*p_1,\\ \displaystyle \frac{dz_1}{dt}=\frac{(k_2-k_3)\beta p_1^*z_1}{a+p_1^*} +\frac{(k_2-k_3)a\beta p_1z_1}{(a+p_1^*)^2}-(D+d_2)z_1.\\ \end{array} \right.$$

Then, (0, 0, 0) is an equilibrium point of (10). Define a positive function

$$V_1=\frac{1}{2}N_1^2+\frac{\sigma _1}{2}p_1^2+\frac{\sigma _2}{2}z_1^2\geqslant 0,$$

where $\sigma _1>0$, $\sigma _2>0$ are to be chosen. Now, calculating the derivative of $V_1$ along the positive solution of system (10) we have

$$\begin{aligned} \frac{dV_1}{dt}\Big |_{(10)}&=N_1\dot{N_1}+\sigma _1p_1\dot{p_1}+\sigma _2z_1\dot{z_1}\\ &=N_1^2(-D-\alpha p_1^*-\alpha p_1)+\sigma _1p_1^2\left[ k_1\alpha (N_1^*+N_1)-(D+d_1) -\frac{a\beta z_1}{(a+p_1^*)^2}-2\upsilon p_1^*\right] \\&\quad +\sigma _2z_1^2 \left( \frac{(k_2-k_3)\beta p_1^*}{a+p_1^*}+\frac{(k_2-k_3) a\beta p_1}{(a+p_1^*)^2}-(D+d_2)\right) +N_1p_1\left( -\alpha N_1^* +m_1d_1+k_1\alpha \sigma _1p_1^*\right) \\&\quad -\frac{\sigma _1\beta p_1^*}{a+p_1^*}p_1z_1+m_2d_2N_1z_1. \end{aligned}$$

Using the inequality

$$\begin{aligned} N_1p_1\leqslant \frac{1}{2}\mu _1N_1^2+\frac{1}{2\mu _1}p_1^2,\quad p_1z_1\leqslant \frac{1}{2}\mu _2p_1^2+\frac{1}{2\mu _2}z_1^2,\quad N_1z_1\leqslant \frac{1}{2}\mu _3z_1^2+\frac{1}{2\mu _3}N_1^2, \end{aligned}$$

we have

$$\begin{aligned} \frac{dV_1}{dt}\Big | _{(10)}&\leqslant N_1^2\left[ -D-\alpha p_1^*-\alpha p_1+\frac{\mu _1}{2}\left( -\alpha N_1^*+m_1d_1+k_1\alpha \sigma _1p_1^*\right) +\frac{m_2d_2}{2\mu _3}\right] +p_1^2\left[ \sigma _1k_1\alpha (N_1^*+N_1)\right. \\&\quad \left. -\,\sigma _1(D+d_1)-\frac{a\sigma _1\beta z_1}{(a+p_1^*)^2}-2\sigma _1\upsilon p_1^*+\frac{1}{2\mu _1}(-\alpha N_1^*+m_1d_1+k_1\alpha \sigma _1p_1^*)-\frac{\mu _2}{2}\frac{\sigma _1\beta p_1^*}{a+p_1^*}\right] \\&\quad +z_1^2\left[ \frac{\sigma _2(k_2-k_3)\beta p_1^*}{a+p_1^*}+\frac{\sigma _2(k_2-k_3)a\beta p_1}{(a+p_1^*)^2}-\sigma _2(D+d_2)-\frac{1}{2\mu _2}\frac{\sigma _1\beta p_1^*}{a+p_1^*}+\frac{\mu _3}{2}m_2d_2\right] . \end{aligned}$$

Set

11 $$\left\{ \begin{array}{ll} \displaystyle \sigma _1=\frac{\alpha N_1^*-m_1d_1}{k_1\alpha p_1^*}-\eta _1,\\ \displaystyle \mu _1=\frac{\alpha N_1^*-m_1d_1-k_1\alpha \sigma _1p_1^*}{2\sigma _1k_1\alpha (N_1^*+N_1)},\\ \displaystyle \mu _2=1,\\ \displaystyle \mu _3=\frac{m_2d_2}{2D},\\ \displaystyle \sigma _2=\frac{\sigma _1 p_1^*(a+p_1^*)}{2a\mu _2(k_2-k_3)p_1}-\eta _2.\\ \end{array} \right.$$

with $\eta _1>0$, $\eta _2>0$. By choosing $\eta _1$, $\eta _2$ properly it is possible to set $\sigma _1$ and $\sigma _2$ such that $\frac{dV_1}{dt}\Big |_{(10)}\leqslant 0$, that is , we can choose $\eta _1$ and $\eta _2$ such that

12 $$\alpha N_1^*-m_1d_1>0,\quad \frac{\sigma _2(k_2-k_3)\beta p_1^*}{a+p_1^*}-\sigma _2(D+d_2)+\frac{\mu _3}{2}m_2d_2<0.$$

So, if (12) holds, $\frac{dV_1}{dt}\Big |_{(10)}\leqslant 0$. $\frac{dV_1}{dt}\Big |_{(10)}=0$ if and only if $(N_1,p_1,z_1)=(0,0,0)$. Thus by Lyapunov–LaSalle invariance principle we obtain the following theorem

#### **Theorem 5**

*Suppose that the equilibrium point*$E_1(N_1^{*},p_1^{*},0)$*of system* (1) *exists. Then it is globally asymptotically stable if* (12) *holds, where*$\sigma _1$, $\sigma _2$, $\mu _1$, $\mu _2$, $\mu _3$*are given by* (11).

Let us consider the transformations $N=N^*+N_2$, $p=p^*+p_2$, $z=z^*+z_2$. With these transformations, the model system (1) reduces to

13 $$\left\{ \begin{array}{ll} \displaystyle \frac{dN_2}{dt}=-DN_2-\alpha (N_2p_2+N^*p_2+N_2p^*)+m_1d_1p_2+m_2d_2z_2,\\ \displaystyle \frac{dp_2}{dt}=k_1\alpha (N_2p_2+N^*p_2+N_2p^*)-(D+d_1)p_2-\frac{a\beta p_2z_2}{(a+p^*)^2}-\frac{a\beta z^* p_2}{(a+p^*)^2}-\frac{\beta p^*z_2}{a+p^*}-2\upsilon p^*p_2,\\ \displaystyle \frac{dz_2}{dt}=\frac{(k_2-k_3)\beta p^*z_2}{a+p^*}+\frac{(k_2-k_3)a\beta p_2z_2}{(a+p^*)^2}+\frac{(k_2-k_3)a\beta z^*p_2}{(a+p^*)^2}-(D+d_2)z_2.\\ \end{array} \right.$$

Then, (0, 0, 0) is an equilibrium point of (13). Define a positive function

$$V_2=\frac{1}{2}N_2^2+\frac{\delta _1}{2}p_2^2+\frac{\delta _2}{2}z_2^2\geqslant 0,$$

where $\delta _1>0$, $\delta _2>0$ are to be chosen. Now, calculating the derivative of $V_2$ along the positive solution of system (13) we have

$$\begin{aligned} \frac{dV_2}{dt}\Big |_{(13)}&=N_2\dot{N_2}+\delta _1p_2\dot{p_2}+\delta _2z_2\dot{z_2}\\ &=N_2^2(-D-\alpha p^*-\alpha p_2)+\delta _1p_2^2\left[ k_1\alpha (N^*+N_2)-(D+d_1)-\frac{a\beta z_2}{(a+p^*)^2}-\frac{a\beta z^*}{(a+p^*)^2}\right. \\&\quad\left. -\,2\upsilon p^*\right] +\delta _2z_2^2 \left( \frac{(k_2-k_3)\beta p^*}{a+p^*}+\frac{(k_2-k_3)a\beta p_2}{(a+p^*)^2}-(D+d_2)\right) +N_2p_2\left( -\alpha N^*+m_1d_1\right. \\&\quad\left. +\,k_1\alpha \delta _1p^*\right) +p_2z_2\left( \frac{\delta _2(k_2-k_3)a\beta z^*}{(a+p^*)^2}-\frac{\delta _1\beta p^*}{a+p^*}\right) +m_2d_2N_2z_2. \end{aligned}$$

Using the inequality

$$\begin{aligned} N_2p_2\leqslant \frac{1}{2}\nu _1N_2^2+\frac{1}{2\nu _1}p_2^2,\quad p_2z_2\leqslant \frac{1}{2}\nu _2p_2^2+\frac{1}{2\nu _2}z_2^2,\quad N_2z_2\leqslant \frac{1}{2}\nu _3z_2^2+\frac{1}{2\nu _3}N_2^2, \end{aligned}$$

we have

$$\begin{aligned} \frac{dV_2}{dt}\Big |_{(13)}&\leqslant N_2^2\left[ -D-\alpha p^*-\alpha p_2+\frac{\nu _1}{2}\left( -\alpha N^*+m_1d_1+k_1\alpha \delta _1p^*\right) +\frac{m_2d_2}{2\nu _3}\right] +p_2^2\left[ \delta _1k_1\alpha (N^*+N_2)\right. \\&\left. \quad -\,\delta _1(D+d_1)-\frac{a\delta _1\beta z_2}{(a+p^*)^2}-\frac{a\delta _1\beta z^*}{(a+p^*)^2}-2\delta _1\upsilon p^*+\frac{1}{2\nu _1}(-\alpha N^*+m_1d_1+k_1\alpha \delta _1p^*)\right. \\&\left. \quad +\,\frac{\nu _1}{2} \left( \frac{\delta _2(k_2-k_3)a\beta z^*}{(a+p^*)^2} -\frac{\delta _1\beta p^*}{a+p^*}\right) \right] +z_2^2 \left[ \frac{\delta _2(k_2-k_3)\beta p^*}{a+p^*}+\frac{\delta _2(k_2-k_3)a\beta p_2}{(a+p^*)^2}-\delta _2(D+d_2)\right. \\&\quad \left. +\,\frac{1}{2\nu _2}\left( \frac{\delta _2(k_2-k_3)a\beta z^*}{(a+p^*)^2}-\frac{\delta _1\beta p^*}{a+p^*}\right) +\frac{\nu _3}{2}m_2d_2\right] . \end{aligned}$$

Set

14 $$\begin{aligned} \left\{ \begin{array}{ll} \displaystyle \delta _1=\frac{\alpha N^*-m_1d_1}{k_1\alpha p^*}-\zeta _1,\\ \displaystyle \nu _1=\frac{\alpha N^*-m_1d_1-k_1\alpha \delta _1p^*}{2\delta _1k_1\alpha (N^*+N_2)},\\ \displaystyle \nu _2=1,\\ \displaystyle \nu _3=\frac{m_2d_2}{2D},\\ \displaystyle \delta _2=\frac{\delta _1 p^*(a+p^*)}{a(k_2-k_3)z^*}-\zeta _2.\\ \end{array} \right. \end{aligned}$$

with $\zeta _1>0$, $\zeta _2>0$. By choosing $\zeta _1$, $\zeta _2$ properly it is possible to set $\delta _1$ and $\delta _2$ such that $\frac{dV_2}{dt}\Big |_{(13)}\leqslant 0$, that is, we can choose $\zeta _1$ and $\zeta _2$ such that

15 $$\alpha N^*-m_1d_1>0,\quad \frac{\delta _2(k_2-k_3)\beta (ap_3+ap^*+p^{*2})}{(a+p^*)^2}-\delta _2(D+d_2)+\frac{\nu _3}{2}m_2d_2<0.$$

So, if (15) holds, $\frac{dV_2}{dt}\Big |_{(13)}\leqslant 0$. $\frac{dV_2}{dt}\Big |_{(13)}=0$ if and only if $(N_2,p_2,z_2)=(0,0,0)$. Thus by Lyapunov–LaSalle invariance principle we obtain the following theorem.

#### **Theorem 6**

*Suppose that the equilibrium point*$E^*(N^{*},p^{*},z^{*})$*of system* (1) *exists. Then it is globally asymptotically stable if condition* (15) *hold, where*$\delta _1$, $\delta _2$, $\nu _1$, $\nu _2$, $\nu _3$*are given by* (14).

### Model (1) with delay

In this section, we discuss the asymptotic stability of coexistence equilibrium and the existence of Hopf bifurcations of the delayed model (1). To simplify the analysis, it is assumed that all the delays are of equal magnitude, i.e. $\tau = \tau _1= \tau _2=\tau _3,$ and $m_2=0$, namely reconversion of dead zooplankton biomass into nutrient is ignored.

We need the following result which was proved in Ruan and Wei (2003) by using Rouches theorem and it is a generalization of the lemma in Dieudonne (1960).

#### **Lemma 1**

*Consider the exponential polynomial*

$$\begin{aligned} P(\lambda , e^{-\lambda \tau _1}, e^{-\lambda \tau _2},\ldots ,e^{-\lambda \tau _m})&= \lambda ^n +p_1^{(0)}\lambda ^{n-1}+p_2^{(0)}\lambda ^{n-2}+\cdots +p_n^{(0)}\\&\quad+ (p_1^{(1)}\lambda ^{n-1}+p_2^{(1)}\lambda ^{n-2}+\cdots +p_n^{(1)})e^{-\lambda \tau _1} +\cdots \\&\quad+(p_1^{(m-1)}\lambda ^{n-1}+p_2^{(m-1)}\lambda ^{n-2}+\cdots +p_n^{(m-1)}) e^{-\lambda \tau _m}, \end{aligned}$$

*where*$\tau _i\ge 0 ( i=1,2,\ldots , m)$*and*$p_j^{(i)} (i=0,1,\ldots , m-1, j=1,2,\ldots , n)$*are constants. As*$( \tau _1, \tau _2,\ldots , \tau _m)$*vary, the sum of the orders of the zeros of*$P(\lambda , e^{-\lambda \tau _1}, e^{-\lambda \tau _2},\ldots ,e^{-\lambda \tau _m})$*on the open right half plane can change only if a zero appears on or crosses the imaginary axis.*

From “Model (1) without delay” section 3.2 we know that the coexistence equilibrium $E^{*}(N^{*},p^{*},z^{*})$ is locally asymptotically stable for $\tau =0$ if (7) holds. For $\tau \ne 0$, the linearization of system (1) at $E^*(N^{*},p^{*},z^{*})$ is

16 $$\begin{aligned} \left\{ \begin{array}{ll} \displaystyle \frac{dN}{dt}=(-D-\alpha p^{*})N(t)-\alpha N^{*}p(t)+m_1d_1p(t-\tau ),\\ \displaystyle \frac{dp}{dt}=k_1\alpha p^{*}N(t)+\left( \frac{\beta p^{*}z^{*}}{(a+p^{*})^{2}}-\upsilon p^{*}\right) p(t)-\frac{\beta p^{*}}{a+p^{*}}z(t),\\ \displaystyle \frac{dz}{dt}=\frac{ak_2\beta z^{*}}{(a+p^{*})^{2}}p(t)-\frac{ak_3\beta z^{*}}{(a+p^{*})^{2}}p(t-\tau ).\\ \end{array} \right. \end{aligned}$$

Then the associated characteristic equation of (16) is

17 $$\begin{aligned} G(\lambda , \tau )=\lambda ^{3}+a_1\lambda ^{2}+a_2\lambda +a_3+e^{-\lambda \tau }(a_4\lambda +a_5)=0, \end{aligned}$$

where $$a_1=D+\alpha p^{*}-\frac{\beta p^{*}z^{*}}{(a+p^{*})^{2}}+\upsilon p^{*}$$, $$a_2= k_1\alpha ^2 N^{*}p^{*}-(D+\alpha p^{*})(\frac{\beta p^{*}z^{*}}{(a+p^{*})^{2}}-\upsilon p^{*})+\frac{a k_2\beta ^2 p^{*}z^{*}}{(a+p^{*})^{3}}$$, $$a_3=(D+\alpha p^{*})\frac{a k_2\beta ^2 p^{*}z^{*}}{(a+p^{*})^{3}}$$, $$a_4=-(\frac{a k_3\beta ^2 p^{*}z^{*}}{(a+p^{*})^{3}}+ k_1\alpha m_1d_1p^{*})$$, $$a_5=-(D+\alpha p^{*})\frac{a k_3\beta ^2 p^{*}z^{*}}{(a+p^{*})^{3}}$$.

In the following, we study the Hopf bifurcation of the coexistence equilibrium. Now for $\tau \ne 0$, if $\lambda =i\omega (\omega >0)$ is a root of $G(\lambda , \tau )=0$, then we have

$$-i\omega ^{3}-a_1\omega ^{2}+a_2\omega i+a_3+[\cos (\omega \tau )-i\sin (\omega \tau )](a_4\omega i+a_5)=0.$$

Separating the real and imaginary parts, we have

18 $$\left\{ \begin{array}{ll} \displaystyle a_5\cos (\omega \tau )+a_4\omega \sin (\omega \tau )=a_1\omega ^{2}-a_3,\\ \displaystyle a_4\omega \cos (\omega \tau )-a_5\sin (\omega \tau )=\omega ^{3}-a_2\omega . \end{array} \right.$$

Adding up the squares of both equations, we obtain

19 $$\omega ^{6}+(a_1^{2}-2a_2)\omega ^{4}+(a_2^{2}-2a_1a_3-a_4^{2}) \omega ^{2}+a_3^{2}-a_5^{2}=0.$$

Denot $r=\omega ^{2}$, then (19) becomes

20 $$r^{3}+b_1r^{2}+b_2r+b_3=0,$$

where $b_1=a_1^{2}-2a_2$, $b_2=a_2^{2}-2a_1a_3-a_4^{2}$ and $b_3=a_3^{2}-a_5^{2}>0$. Let

21 $$g(r)=r^{3}+b_1r^{2}+b_2r+b_3.$$

By the ideal of Li and Wei (2005), Ruan and Wei (2001), Song and Wei (2004), in what follows, we study the distribution of the zeros of (20). From $g(0)=b_3=a_3^{2}-a_5^{2}>0$ we can easily get the following lemma.

#### **Lemma 2**

*Equation* (20) *has at least one negative real root.*

#### **Lemma 3**

*If*$\Delta =b_1^{2}-3b_2\le 0$, *then* Eq. (20) *has no positive roots.*

#### *Proof*

From (21) we have $\frac{dg(r)}{dr}=3r^{2}+2b_1r+b_2$. Set

22 $$3r^{2}+2b_1r+b_2=0.$$

Then the roots of Eq. (22) can be expressed as

23 $$r_{1,2}=\frac{-2b_1\pm \sqrt{4b_1^{2}-12b_2}}{6}= \frac{-b_1\pm \sqrt{b_1^{2}-3b_2}}{3}=\frac{-b_1\pm \sqrt{\Delta }}{3}.$$

If $\Delta \le 0$, then (22) has no real roots or exists one root. So the function *g*(*r*) is monotone increasing with *r*. Therefore, Eq. (20) has no positive real roots due to $g(0)=b_3>0$. $\square$

Obviously, if $\Delta >0$, then $r_1=\frac{-b_1+\sqrt{\Delta }}{3}$ is the local minimum of *g*(*r*). Thus, we get the following result.

#### **Lemma 4**

*Equation* (20) *has positive roots if and only if*$r_1>0$*and*$g(r_1)\le 0$.

#### *Proof*

The sufficiency is obvious. We only need to prove the necessity. Otherwise, we assume that either $r_1\le 0$ or $r_1>0$ and $g(r_1)>0$. Since g(r) is increasing for $r\ge r_1$ and $g(0)=b_3>0$, *g*(*r*) has no positive real zeros for $r_1\le 0$. If $r_1>0$ and $g(r_1)>0$, since $r_2=\frac{-b_1-\sqrt{\Delta }}{3}$ is the local maximum value, it gives that $g(r_1)<g(r_2)$. Hence, by $g(0)=b_3>0$ we obtain that *g*(*r*) has no positive real zeros. This completes the proof. $\square$

From above discussion, we get the following lammas.

#### **Lemma 5**

*Suppose that*$r_1$*is defined by* (23).

*(a)*: *Eq.* (20) *has at least one negative real root.*
*(b)*: *If*$\Delta =b_1^{2}-3b_2\le 0$, *then Eq.* (20) *has no positive roots*.
*(c)*: *Eq.* (20) *has positive roots if and only if*$r_1>0$*and*$g(r_1)\le 0$.

Assume that Eq. (20) has positive roots. Without loss of generality, we suppose that it has two positive roots, denoted by $u_1$, $u_2$, respectively. Then (19) has two positive roots, say

$$\begin{aligned} \omega _1=\sqrt{u_1},\quad \omega _2=\sqrt{u_2}. \end{aligned}$$

By (18) we have

$$\cos (\omega _k\tau )=\frac{a_4\omega _k^{4}+(a_1a_5-a_2a_4)\omega _k^{2}-a_3a_5}{a_4^{2}\omega _k^{2}+a_2^{2}},\quad k=1,2.$$

Let

$$\begin{aligned} \tau _k^{j}= {\left\{ \begin{array}{ll} \frac{1}{\omega _k}\left( \arccos \left( \frac{a_4\omega _k^{4}+(a_1a_5-a_2a_4)\omega _k^{2}-a_3a_5}{a_4^{2}\omega _k^{2}+a_2^{2}}\right) +2j\pi \right) &\quad for \; \sin \left( \omega _k\tau _k^{j}\right) >0,\\ \frac{1}{\omega _k}\left( 2\pi -\arccos \left( \frac{a_4\omega _k^{4}+(a_1a_5-a_2a_4)\omega _k^{2}-a_3a_5}{a_4^{2}\omega _k^{2}+a_2^{2}}\right) +2j\pi \right) &\quad for \; \sin \left( \omega _k\tau _k^{j}\right) <0, \end{array}\right. } \end{aligned}$$

where $$k=1,2;j=0,1,2,....$$

Then $\pm i\omega _k$ is a pair of purely imaginary roots of (17), $\tau =\tau _k^{j}$. define

$$\tau _0=\tau _{k_o}^{0}=\min \limits _{k\in {1,2}}\{\tau _{k}^{0}\},\omega _0=\omega _{k_0}.$$

Therefore, applying Lemmas 1 and 5 to (17), we obtain the following lemma.

#### **Lemma 6**

*Suppose that the inequality* (7) *holds. Then we have*

*(a)*: *If*$\Delta =b_1^{2}-3b_2\le 0$, *then all roots of equation* (17) *have negative real parts for all*$\tau \ge 0$.
*(b)*: *If*$\Delta =b_1^{2}-3b_2>0$, $r_1>0$*and*$g(r_1)\le 0$, *then Eq.* (17) *has a pair of imaginary roots*$\pm i\omega _0$. *Furthermore, if*$\tau \in [0, \tau _0)$, *then all roots of equation* (17) *have negative real parts.*

Let $\lambda (\tau )=\xi (\tau )+i\omega (\tau )$ be the root of (17) near $\tau =\tau _0$ satisfying $\xi (\tau _0)=0$, $\omega (\tau _0)=\omega _0$. Let $r_0=\omega _0^{2}$. Then we have the following transversality condition.

#### **Lemma 7**

*Suppose*$g'(r_0)\ne 0$. *If the conditions of Lemma* 6*(b) are satisfied, then*$\frac{dRe\lambda (\tau _0)}{d\tau }\ne 0$, $\frac{dRe\lambda (\tau _0)}{d\tau }$*and*$g'(r_0)$*have the same sign.*

#### *Proof*

Differentiating (17) with respect to $\tau$, we obtain

$$\begin{aligned} \frac{d\lambda }{d\tau }\left[ 3\lambda ^{2}+2a_1\lambda +a_2-\tau (a_4\lambda +a_5) e^{-\lambda \tau }+a_4e^{-\lambda \tau }\right] -e^{-\lambda \tau }\lambda (a_4\lambda +a_5)=0. \end{aligned}$$

It follows that

$$\begin{aligned} \frac{d\lambda }{d\tau }=\frac{e^{-\lambda \tau }\lambda (a_4\lambda +a_5)}{3\lambda ^{2}+2a_1\lambda +a_2-\tau (a_4\lambda +a_5)e^{-\lambda \tau }+a_4e^{-\lambda \tau }}. \end{aligned}$$

Then

$$\begin{aligned} \left[ \frac{d\lambda }{d\tau }\right] ^{-1}= \frac{(3\lambda ^{2}+2a_1\lambda +a_2)e^{\lambda \tau }+a_4}{(a_4\lambda +a_5)\lambda }-\frac{\tau }{\lambda }. \end{aligned}$$

From (19), we have

$$\begin{aligned} Re\left[ \left( \frac{d\lambda }{d\tau }\right) ^{-1}\right] _{\tau =\tau _0}&= Re\left[ \left( \frac{d\lambda }{d\tau }\right) ^{-1}\right] _{\lambda =i\omega _0}\\&= Re\left[ \frac{\left( 3(i\omega _0)^{2}+2a_1i\omega _0+a_2\right) e^{i\omega _0\tau }+a_4}{(a_4i\omega _0+a_5)i\omega _0}\right] \\&= \frac{\omega _0^{2}}{a_4^{2}\omega _0^{4}+a_5^{2}\omega _0^{2}} \left[ 3\omega _0^{4}+(2a_1^{2}-4a_2)\omega _0^{2}+a_2^{2}-2a_1a_3-a_4^{2}\right] \\&= \frac{1}{a_4^{2}\omega _0^{2}+a_5^{2}}g^{'}(r_0). \end{aligned}$$

Thus, we have

$$\begin{aligned} sign\left[ \frac{dRe\lambda (\tau _0)}{d\tau }\right] = sign{\left[ \frac{dRe\lambda (\tau _0)}{d\tau }\right] ^{-1}}= sign\left[ \frac{1}{a_4^{2}\omega _0^{2}+a_5^{2}}g^{'}(r_0)\right] . \end{aligned}$$

Since $\frac{1}{a_4^{2}\omega _0^{2}+a_5^{2}}>0$, we conclude that $\frac{dRe\lambda (\tau _0)}{d\tau }\ne 0$, $\frac{dRe\lambda (\tau _0)}{d\tau }$ and $g^{'}(r_0)$ have the same sign. This completes the proof. $\square$

#### **Theorem 7**

*Suppose that the inequality* (7) *holds.*

*(a)*: *If*$\Delta =b_1^{2}-3b_2\le 0$, *then the coexistence equilibrium*$E^{*}$*of system* (1) *is asymptotically stable for all*$\tau \ge 0$.
*(b)*: *If*$\Delta =b_1^{2}-3b_2>0$, $r_1=\frac{-b_1+\sqrt{\Delta }}{3}>0$*and*$g'(r_0)<0$*hold, then system* (1) *at the equilibrium*$E^{*}$*is asymptotically stable for*$\tau \in [0,\tau _0)$, *and unstable when*$\tau >\tau _0$. *System* (1) *undergoes a Hopf bifurcation at*$E^{*}$*when*$\tau =\tau _0$.

## Numerical simulation

To substantiate analytical findings a set of hypothetical parameter values have been considered for numerical simulation (see Table 1). Most of the parameters in Table 1 used by authors in Chattopadhayay et al. (2002) and Fan et al. (2013).

**Table 1:** Parameter values used in numerical simulation

Parameters	Symbols	Values
Dilution rate	D	$0.4\;(1 \,\hbox {day}^{-1})$
Constant input of nutrient concentration	$N_0$	40 (mg $\hbox {dm}^{-1}$ )
Nutrient uptake rate for the phytoplankton	$\alpha$	$0.7\;(1\,\hbox {day}^{-1})$
Maximum zooplankton ingestion rate	$\beta$	$0.6\;(1\,\hbox {day}^{-1})$
Conversion factor from death phytoplankton	$m_1$	0.8
Conversion factor from death zooplankton	$m_2$	0.5
Natural death rate of phytoplankton	$d_1$	$0.025\;(\hbox {day}^{-1})$
Natural death rate of zooplankton	$d_2$	$0.02\;(\hbox {day}^{-1})$
Conversion factor from nutrient to phytoplankton	$k_1$	0.9677
Conversion factor from phytoplankton to zooplankton	$k_2$	0.9661
Toxin-production rate	$k_3$	$0.0186\;(\hbox {day}^{-1})$
Half-saturation coefficient	*a*	$2\;(\hbox {mg}\,\hbox {dm}^{-1})$
Intra-specific competition coefficient	$\upsilon$	0.1

**Fig. 1:**
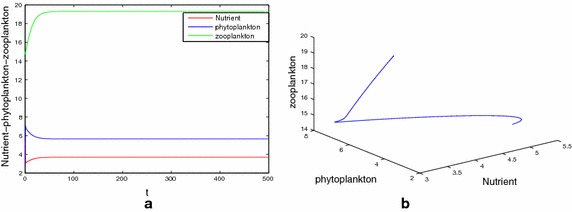
**a**, **b** The asymptotical stability of the coexistence equilibrium $E^*(3.696,5.6566,19.3071)$ with $\tau =1$. Here, initial value is (5, 3, 15 )

**Fig. 2:**
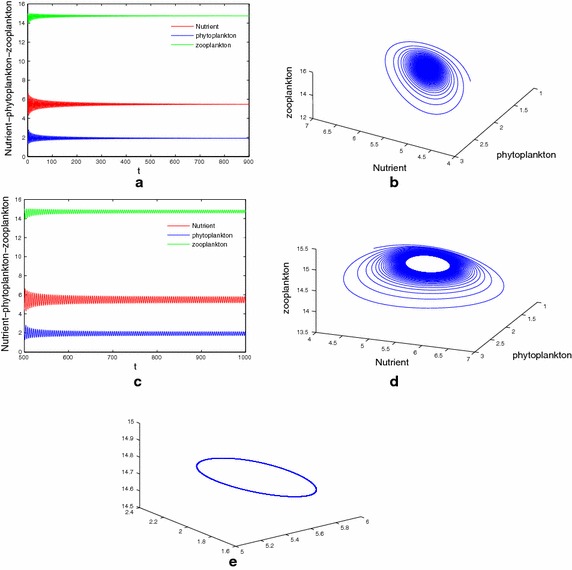
**a**, **b** The asymptotical stability of the coexistence equilibrium $E^*(5.48181.917314.7487)$ with $\tau =1<\tau _0$. **c**–**e** Coexistence equilibrium $E^*$ loses its stability when $\tau =1.9>\tau _0$. Stable periodic solution arising from Hopf bifurcation at $\tau =\tau _0$. Here $\tau _0=1.7657$

First, we consider the special case of system (1), that is, $\tau _1=\tau _2=\tau _3=\tau$ and $m_2=0$. In order to verify the results of Theorem 7, we consider $\tau$ as bifurcation parameter and for case (a) taken parameters in Table 1. It is easy to compute that $\Delta =-159.1443<0.$ Our numerical simulations show that for all $\tau \ge 0$, interior equilibrium $E^*(3.696,5.6566,19.3071)$ is stable. Figure 1 shows the simulation result for the system (1) with $\tau = 1$. For case (b) of Theorem 7, we take parameters as $D=0.3(1\,\hbox {day}^{-1})$, $N_0=26.4\,\hbox {mg}\,\hbox {dm}^{-1}$, $a=1.5\,\hbox {mg}\,\hbox {dm}^{-1}$ and other parameters the same as that in Table 1. A direct computation gives $\Delta =4.5990121>0$, $r_1=2.07198534>0$ and $g^{'}(r_0)=-0.0728333887\ne 0$ holds. After calculations we find the minimum value of the delay parameter ‘$\tau$’ for system (1) for which the stability behaviour changes and the first critical values are given by $\tau _0=1.7657$, such that $E^*(5.4818,1.9173,14.7487)$ is locally stable for $\tau \in [0,1.7657)$ and is unstable for $\tau >\tau _0$. From our analytical findings we have seen that $E^*$ is locally asymptotically stable for $\tau < \tau _0$. Figure 2 shows the simulation result for system (1) with $\tau = 1 < \tau _0$. Interior equilibrium point looses its stability as $\tau$ passes through its critical value $\tau = \tau _0$ and a Hopf bifurcation occurs. A periodic solution is depicted in Fig. 2d, e.

**Fig. 3:**
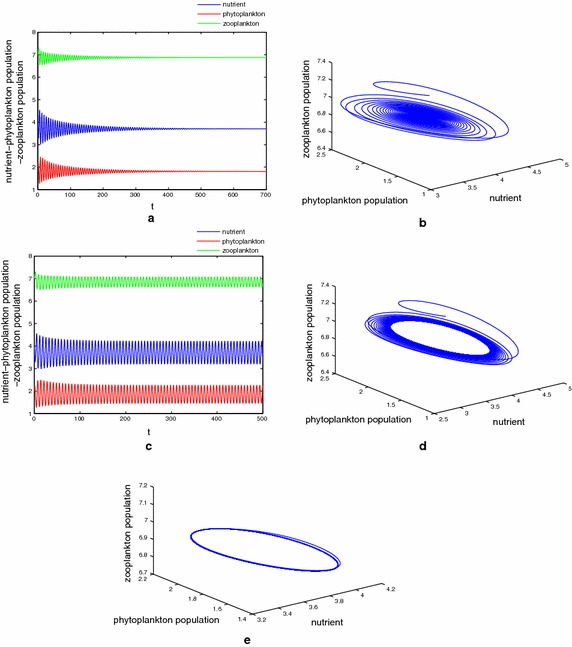
**a**, **b** The asymptotical stability of the coexistence equilibrium $E^*(5.48181.917314.7487)$ for $(\tau _1, \tau _2, \tau _3)$ with $\tau _1=1<\tau _1^0$. **c** Coexistence equilibrium $E^*$ loses its stability at $(\tau _1, \tau _2, \tau _3)$ with $\tau _1=4>\tau _1^0$. Stable periodic solution arising from Hopf bifurcation at $(\tau _1, \tau _2, \tau _3)$ with $\tau _1=\tau _1^0$. **d**, **e** Stable limit cycle is observed at $(\tau _1, \tau _2, \tau _3)$ with $\tau _1=4.2$. Here $\tau _2=1, \tau _3=2$ and $\tau _1^0=3.9465$

**Fig. 4:**
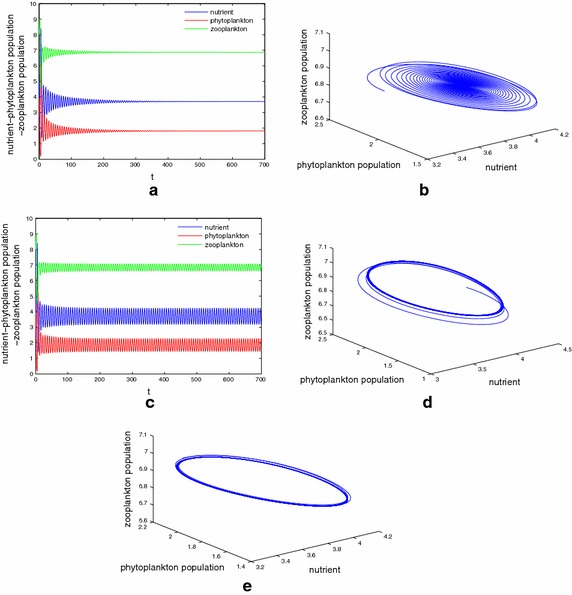
**a**, **b** The coexistence equilibrium $E^*(5.48181.917314.7487)$ becomes stable for $(\tau _1, \tau _2, \tau _3)$ with $\tau _1=8<\tau _1^1$. **c** Coexistence equilibrium $E^*$ loses its stability at $(\tau _1, \tau _2, \tau _3)$ with $\tau _1=11.56>\tau _1^1$. Stable periodic solution arising from Hopf bifurcation at $\tau _1=\tau _1^1$. **d**, **e** Stable limit cycle is observed at $(\tau _1, \tau _2, \tau _3)$ with $\tau _1=12$. Here $\tau _1^1=10.6156$

Next, We present some numerical results on the case of system (1) that $\tau _1\ne \tau _2\ne \tau _3$ and $m_2\ne 0$. Take $D=0.38 1\,\hbox {day}^{-1}$, $N_0=15\,\hbox {mg}\,\hbox {dm}^{-1}$, $k_3=0.1\,\hbox {day}^{-1}$, $a=1\,\hbox {mg}\,\hbox {dm}^{-1}$, $\upsilon =0.009$ and other parameters the same as that in Table 1. With the help of this parameter set we obtain the interior equilibrium as $E^*(3.7040, 1.8108, 6.8715)$. Let us fix $\tau _2=1, \tau _3=2$ and gradually increase the value of $\tau _1$. After some calculations one can find the minimum value of the delay parameter “$\tau _1$” for the model system (1) for which the stability behaviour changes and the first critical values are given by $\tau _1^0=3.9465, \tau _1^1=6.7592$, such that $E^*(3.7040, 1.8108, 6.8715)$ is stable for $\tau _1\in [0, 3.9465)$ and unstable for $\tau _1\in [3.9465, 6.759)$. Figure 3 shows the simulation result for the model system (1) with $\tau _1 = 1 <\tau _1^0$. Interior equilibrium point looses its stability as $\tau _1$ passes through its critical value $\tau _1 =\tau _1^0$ and a Hopf bifurcation occurs, a stable Hopf-bifurcating periodic solution is depicted in Fig. 3d, e. The equilibrium point $E^*(3.7040, 1.8108, 6.8715)$ remains locally asymptotically stable whenever the delay parameter lies in the range (6.759, 10.6156). $E^*(3.7040, 1.8108, 6.8715)$ again switches from stability to instability as $\tau _1$ passes through $\tau _1 =10.6156$ and an unstable solution for the model system (1) is shown in Fig. 4. The numerical simulations we have done here illustrate the stable periodic solution arising from Hopf bifurcation at $\tau _1^0=3.9465, \tau _1^1=6.759$ and $\tau _1^2=10.6156$, respectively, and the switching of stability that occurs as the magnitude of the delay parameter increases gradually. For the above set of parameter values, when fixing $\tau _1, \tau _3$ and varying the value of $\tau _2$ or fixing $\tau _1, \tau _2$ and varying $\tau _3$ , the dynamical behavior of the system (1) explored by numerical simulation are the same as above, so we omit it here.

## Conclusions and discussion

In the present analysis, we have proposed and analyzed a three component model consisting of nutrient, phytoplankton and zooplankton. It is assumed that the grazing on phytoplankton , zooplankton growth rate and the zooplankton mortality due to the toxin phytoplankton are Holling type II forms. According to the facts that reconversion of dead biomass into nutrient is not an instantaneous process, but is mediated by some time lag required, and the toxin liberation by the phytoplankton species also need time period, our model in present paper incorporate delayed nutrient recycling and delayed toxic liberation. In comparison with literatures (Fan et al. 2013; Das and Ray 2008), the model (1) in this paper is more general and realistic.

In the absence of the time delay, the dynamical behavior of system (1) was studied extensively around all feasible equilibria. Conditions were also derived both for the local and global stability of the system at all possible equilibria. Theorem 4 indicates that if the conversion rate from nutrient to phytoplankton and phytoplankton to zooplankton lower than certain values, then plankton will extinct. This result is consistent with real ecosystem. Theorem 5 shows that a high concentration of the input nutrient (together with a high mortality rate of the zooplankton population) will cause eradication of the zooplankton. Theorem 6 reveals that low values of mortality rate of the both phytoplankton and zooplankton population ensures coexistence of the plankton. Thus, the concentration of the input nutrient, the mortality rate of the plankton plays a major role in controlling the local and global dynamics of the basic model around the various stationary states.

Next we have studied the model with discrete delay in the term modeling plankton recycling and the term of toxin liberation. Numerically it is shown that the behavior of the system around the interior equilibrium depends on the time delay. When we fix time delay $$\tau _i, \tau _j$$ and gradually increase the value of $$\tau _l$$$$(i\ne j\ne l, i.j.l=1,2,3)$$, the numerical simulations which we have performed show that there are threshold limit $$\tau _l^k (l=1,2,3, k=1,2,\ldots )$$ such that as the time delay crosses the threshold value $$\tau _l^k$$, the delayed nutrient–plankton system enters into a Hopf bifurcation and we have a periodic orbit around the coexisting equilibrium point $$E^*$$. The interior equilibrium point $$E^*$$ is stable whenever $$\tau \in [0, \tau _l^1)\cup [\tau _l^2, \tau _l^3)\cup \cdots$$ and unstable for $$\tau \in [\tau _l^1, \tau _l^2 )\cup [\tau _l^3, \tau _l^4, )\cup \cdots , l=1,2,3$$. This phenomenon is known as switching of stability which arises for our model system. The most interesting as well as mathematically important results we have presented in this paper is the stability criteria for the Hopf-bifurcating periodic solution by considering the discrete time lag $$\tau$$ as bifurcation parameter. These findings demonstrate the delayed effect of plankton and the cyclic nature of blooms in this nutrient–plankton system. This is one of the most important findings of our analysis. In their analysis, Fan et al. (2013) and Das and Ray (2008) were unable to exhibit the periodic nature of blooms by considering non-delayed nutrient–plankton system. From Figs. 3c and 4c we find the solutions oscillate around $$E^*$$. Figures 3c and 4c show that the plankton system can occurs the peak phenomenon, which corresponds to blooms, and also occurs the valley effect, which corresponds the low values of phytoplankton. Our mathematical and numerical results provide certain threshold values for the delay parameters for which we can maintain a stable situation for all the species and can control bloom dynamics.

Now let us make a comparison with result of previous studies and present study. Fan et al. (2013) investigated a nutrient–plankton system with nutrient recycling from dead plankton, but the time required to regenerate nutrient from dead organic is neglected. Besides, the effects of the toxin which produced by phytoplankton did not take account into their model. Sharma et al. (2014) studied a nutrient–toxin phytoplankton–zooplankton model with nutrient recycling, but there is only nutrient recycling from dead phytoplankton and the recycling assumed to be instantaneous. The model studied by Fan et al. (2013) has an unique interior equilibrium $$E_2$$ under the condition $$N_0>N_1+\bar{N}$$, which also ensures local asymptotic stability of the interior equilibrium $$E_2$$. Our results shows that conditions $$\frac{ak_1\alpha m_2d_2}{\beta }<D<a\alpha$$ and $$0<p^*<p^*_1$$ ensure the existence of the interior equilibrium $$E^*$$. But for locally asymptotically stability of the $$E^*$$, we need condition (7). Therefore, here the coexistence equilibrium in this setting possesses more restrictive existence and stability condition, since they involve the intra-specific competition parameter $$\upsilon$$ and toxin liberation parameter $$k_3$$, see Theorems 2 and 3. The local and global stability of the interior equilibrium have not studied by Sharma et al. (2014). While the globally asymptotically stability of the interior equilibrium $$E_2$$ have been proved by Fan et al. (2013) only for the special case of model (1) (with $$m_i=0, i=1,2)$$. In this paper, we obtained sufficient conditions which ensures for the interior equilibrium of the model (1.2) to be globally asymptotically stable. This can be seen as one of the novelty of this paper. Moreover, the results obtained by Fan et al. (2013) indicate that the concentration of the input nutrient $$N_0$$ and the initial conditions of the nutrient–plankton model are the two important factors on the dynamics of the system behavior. But here, our results obtained in this paper indicate that except for concentration of the input nutrient and initial values of the system (1.2), the intra-specific parameter and toxin liberation parameter also affect the dynamical properties of the model (1.2). Comparing with paper (Sharma et al. 2014), a Hopf-bifurcation arises also at the interior equilibrium, but the conditions for its occurrence here at $$E^*$$ are more restrictive, involving also the intra-specific competition, recycling of the zooplankton and toxin liberation. Besides, differently from literatures (Fan et al. 2013; Sharma et al. 2014), our numerical investigations show that the nutrient recycling delays can induce stability switches, such that the interior equilibrium switches from stable coexistence equilibrium to stale periodic orbits, to stable coexistence equilibrium again and so on(see Figs. 3, 4). This phenomenon is ecologically important and especially can lead to potentially dramatic shifts to the system dynamics. In biological terms, our finding has ecological significance in the estuarine system. There are jungles and forest adjacent to the estuary, which are the main source of productivity. The nutrients come from the litterfall which can be decomposed after a period of time. The tide not only collects the nutrient from the litters but also mixes them into the estuarine water (Wang and Wang (2007)). Our research indicates that delays in the decomposition of litterfall cause destabilization of this system.

Unfortunately, we cannot give a complete mathematical analysis of the asymptotic stability of the positive equilibrium $E^*$ for model (1) with different delay, i.e., $\tau _1\ne \tau _2\ne \tau _3,$ and $m_2\ne 0$. We shall leave the problems as future work.

## References

[CR1] Abdllaoui AE, Chattopadhyay J, Arino O (2002). Comparisons, by models, of some basic mecha nisms acting on the dynamics of the zooplankton-toxic phytoplankton systems. Math Models Methods Appl Sci.

[CR2] Aiello WG, Freedman HI (1990). A time delay model of single species growth with stage-structure. Math Biosci.

[CR3] Angelis DL (1992). Dynamics of nutrient recycling and food webs.

[CR4] Barton AD, Dutkiewicz S (2010). Patterns of diversity in marine phytoplankton. Science.

[CR5] Beretta E, Bischi GI, Solimano F (1990). Stability in chemostat equations with delayed recycling. J Math Biol.

[CR6] Bischi GI (1992). Effects of time lags on transient characteristics of a nutrient recycling model. Math Biosci.

[CR7] Chakarborty S, Roy S, Chattopadhyay J (2008). Nutrient-limited toxin producing and the dynamics of two phytoplankton in culture media: a mathematical model. J Ecol Model.

[CR8] Chakraborty K, Dasb K (2015). Modeling and analysis of a two-zooplankton one-phytoplankton system in the presence of toxicity. Appl Math Model.

[CR9] Chattopadhyay J, Sarkkar RR, Abdllaoui A (2002). A delay differential equation model on harmful algal blooms in the presence of toxic substances. IMA J Math Appl Med Biol.

[CR10] Chattopadhayay J, Sarkarw RR, Mandalw S (2002). Toxin-producing plankton may act as a biological control for planktonic blooms—field study and mathematical modelling. J Theor Biol.

[CR11] Chatterjee A, Pal S, Chatterjee S (2011). Bottom up and top down effect on toxin producing phytoplankton and its consequence on the formation of plankton bloom. Appl Math Comput.

[CR12] Chen Y, Yu J, Sun C (2007). Stability and Hopf bifurcation analysis in a three-level food chain system with delay. Chaos Solitons Fractals.

[CR13] Cooke K, Grossman Z (1982). Discrete delay, distributed delay and stability switches. J Math Anal Appl.

[CR14] Chowdhury T, Roy S, Chattopadhyay J (2008). Modeling migratory grazing of zooplankton on toxic and non-toxic phytoplankton. Appl Math Comput.

[CR15] Das K, Ray S (2008). Effect of delay on nutrient cycling in phytoplankton–zooplankton interactions in estuarine system. Ecol Model.

[CR16] Dieudonne J (1960). Foundations of modern analysis.

[CR17] Edwards AM, Brindley J (1999). Zooplankton mortality and the dynamical behaviour of plankton population model. Bull Math Biol.

[CR18] Fan A, Han P, Wang K (2013). Global dynamics of a nutrient–plankton system in the water ecosystem. Appl Math Comput.

[CR19] Ghosh D, Sarkar AK (1998). Stability and oscillations in resource-based model of two interacting species with nutrient recycling. Ecol Model.

[CR20] Hassard B, Kazarinoff N, Wan YH (1981) Theory and applications of Hopf bifurcation. In: London mathematical society lecture notes, series, 41. Cambridge University Press, Cambridge

[CR21] He X, Ruan S (1998). Global stability in chemostat-type plankton models with delayed nutrient recycling. J Math Biol.

[CR22] Holling CS (1965). The functional response of predators to prey density and its role in mimicry and population regulation. Mem Entromol Soc Can.

[CR23] Huppert A, Olinkly R, Stone L (2004). Bottom-up excitable models of phytoplankton blooms. Bull Math Biol.

[CR24] Jana S, Chakraborty M, Chakraborty K (2012). Global stability and bifurcation of time delayed prey–predator system incorporating prey refuge. Math Comput Simul.

[CR25] Jang SRJ, Baglama J, Rick J (2006). Nutrient–phytoplankton–zooplankton models with a toxin. Math Comput Model.

[CR26] Khare S, Dhar J, Misra OP (2010). Role of toxin producing phytoplankton on a plankton ecosystem. Nonlinear Anal Hybrid Syst.

[CR27] Li X, Wei J (2005). On the zeros of a fourth degree exponential polynomial with applications to a neural network model with delays. Chaos Solitions Fractals.

[CR28] Ludwig D (1978). Qualitative analysis of an insect outbreak system: the spruce budworm and forest. J Anal Ecol.

[CR29] Ma Z (1996). Mathematical modelling and study of population biology.

[CR30] Mukhopadhyay B, Bhattacharyya R (2010). Kolkata. Modeling the role of constant and time varying recycling delay on an ecological food chain. Appl Math.

[CR31] Nisbet RM, Mckinstry J, Gurney WSC (1983). A “strategic” model of material cycling in a closed ecosystem. Math Biosci.

[CR32] Odum PE (1971). Fundamentals of ecology.

[CR33] Pal S, Chatterjee S, Chattopadhyay J (2007). Role of toxin and nutrient for the occurrence and termi nation of plankton bloom-results drawn from field observations and a mathematical model. J Biosyst.

[CR34] Pardo O (2000). Global stability for a phytoplankton–nutrient system. J Biol Syst.

[CR35] Rehim M, Imran M (2012). Dynamical analysis of a delay model of phytoplankton–zooplankton interaction. Appl Math Model.

[CR36] Rose KA (2012). End-to-end models for marine ecosystems: are we on the precipice of a significant advance or just putting lipstick on a pig?. Sci Mar.

[CR37] Ruan S (1995). The effect of delays on stability and persistence in plankton models. Nonlinear Anal.

[CR38] Ruan S, Ardito A, Ricciardi P, DeAngelis D (2007). Coexistence in competition models with density dependent mortality. C R Biol.

[CR39] Ruan S, Wei J (2003). On the zeros of transcendental function with applications to stability of delay differential equations. Dyn Contin Discrete Impuls Syst.

[CR40] Ruan S, Wei J (2001). On the zeros of a third degree exponential polynomial with applications to a delayed model for the control of testosterone secretion. IMA J Math Appl Med Biol.

[CR41] Ruan S (2001). Oscillations in plankton models with nutrient recycling. J Theor Biol.

[CR42] Ruan S (1993). Persistence and coexistence in zooplankton–phytoplankton–nutrient models with in instantaneous nutrient recycling. J Math Biol.

[CR43] Saha T, Bandyopadhyay M (2009). Dynamical analysis of toxin producing phytoplankton–zooplankton interactions. Nonlinear Anal RWA.

[CR44] Sharma A, Sharma AK, Agnihotri K (2014). The dynamic pf plankton–nutrient interaction with delay. Appl Math Comput.

[CR45] Sarkara RR, Palb S, Chattopadhyayc J (2005). Role of two toxin-producing plankton and their effect on phytoplankton–zooplankton system—a mathematical study supported by experimental find ings. BioSystems.

[CR46] Smayda T (1997). What is a bloom? A commentary. Limnol Oceaonogr.

[CR47] Song Y, Wei J (2004). Bifurcation analysis for Chen’s system with delayed feedback and its application to control of chaos. Chaos Solitons Fractals.

[CR48] Song Y, Han M, Peng Y (2004). Stability and Hopf bifurcations in a competitive Lotka–Volterra system with two delays. Chaos Solitons Fractals.

[CR49] Upadhyay RK, Chattopadhyay J (2005). Chaos to order: role of toxin producing phytoplankton in aquatic systems. J Nonlinear Anal Model Control.

[CR50] Wang Y, Wang HB, Jiang WH (2014). Stability switches and global Hopf bifurcation in a nutrient–plankton model. Nonlinear Dyn.

[CR51] Wang WQ, Wang M (2007). The Mangroves of China.

[CR52] Yunfei Lv, Jianzhi Cao, Juan Song, Rong Yuan, Yongzhen Pei (2014). Global stability and Hopf-bifurcation in a zooplankton–phytoplankton model. Nonlinear Dyn.

[CR53] Zhang TR, Wang WD (2012). Hopf bifurcation and bistability of a nutrient–phytoplankton–zooplankton model. Appl Math Model.

